# Sustainable Production and Characterization of Eumelanin from Organically Cultivated *Mucuna ceniza* Seeds: A High-Performance Biomaterial for Optoelectronic Applications

**DOI:** 10.3390/ijms262110298

**Published:** 2025-10-23

**Authors:** Pedro Arturo Herrera-Herrera, Ana Lilia Hernández-Orihuela, Alejandra Renteria-Salcedo, Dulce María Palmerín-Carreño, Alicia Huazano-García, Danae Carrillo-Ocampo, Miguel Angel Ramos-Valdovinos, Agustino Martínez-Antonio

**Affiliations:** 1 Departamento de Química, Centro Universitario de Ciencias Exactas e Ingenierías, Universidad de Guadalajara, Blvd. Marcelino Garcia Barragan 1421, Guadalajara C.P. 44430, Mexico; pedroa.herrera@academicos.udg.mx; 2 Evogenia, 5 de mayo 517, Guanajuato C.P. 36500, Mexico; lilia@biosintetica.mx; 3 Centro Universitario de Ciencias Exactas e Ingenierías, Universidad de Guadalajara, Blvd. Marcelino Garcia Barragan 1421, Guadalajara C.P. 44430, Mexico; ale.renteria.salcedo@gmail.com; 4 Facultad de Química, Universidad Autónoma de Querétaro, Campus Pedro Escobedo, Pedro Escobedo, Querétaro C.P. 76010, Mexico; dulce.palmerin@uaq.mx; 5 Departamento de Biotecnología y Bioquímica, Centro de Investigación y de Estudios Avanzados del IPN-Unidad Irapuato, Guanajuato C.P. 36824, Mexico; alicia.huazano@cinvestav.mx; 6 SECIHTI, Av. Insurgentes Sur 1582, Ciudad de México C.P. 03940, Mexico; 7 Departamento de Ingeniería Genética, Centro de Investigación y de Estudios Avanzados del IPN-Unidad Irapuato, Guanajuato C.P. 36824, Mexico; danae.carrillo@cinvestav.mx; 8 Laboratorio de Ingeniería Biológica, Departamento de Ingeniería Genética, Centro de Investigación y de Estudios Avanzados del IPN-Unidad Irapuato, Guanajuato C.P. 36824, Mexico; miguel.ramos@cinvestav.mx

**Keywords:** natural products, eumelanin, *Mucuna ceniza*, analytical characterization, spectroscopic analysis, biotransformation, L-DOPA, sustainable cultivation, optoelectronics, green extraction, antioxidant properties

## Abstract

Natural melanins represent an emerging class of bio-based materials with exceptional properties for advanced technological applications. This study presents a comprehensive analytical characterization of eumelanin produced from organically cultivated *Mucuna ceniza* seeds through sustainable biotechnological processes. A high-quality L-DOPA extract containing 56% *w*/*w* L-DOPA was first obtained using green extraction protocols with organic acids, followed by lyophilization. Then, optimized stirred-tank bioreactor conditions achieved remarkable melanin production rates of 1526.23 ± 10.78 mg L^−1^ h^−1^ with complete L-DOPA conversion, yielding 9.5 g/L of purified eumelanin. Spectroscopic characterization using UV-visible, FTIR, Raman, and NMR spectroscopy confirmed the authentic eumelanin structure, characterized by a characteristic absorption at 225 nm, diagnostic FTIR bands, Raman signatures at 1380 and 1580 cm^−1^, and NMR peaks. The elemental composition (C: 48.04%, H: 6.14%, N: 11.85%, O: 33.94%) classified the pigment as eumelanin, with an inferred empirical formula of C_48_H_74_N_10_O_25_. This melanin has already demonstrated practical utility in optoelectronic applications. By harnessing the unique biocatalytic potential of organically grown *Mucuna ceniza*, this study validates a green, high-yield production platform for eumelanin, paving the way for its commercially viable application in advanced functional materials.

## 1. Introduction

Natural products continue to serve as invaluable sources of bioactive compounds and functional materials, with melanins representing one of the most ubiquitous and structurally complex biopolymers found in nature [1]. These dark pigments, distributed across diverse biological kingdoms from bacteria to mammals, exhibit remarkable physicochemical properties that have attracted increasing attention for biotechnological and industrial applications [2,3]. The growing interest in sustainable alternatives to synthetic materials has positioned natural melanins as promising candidates for advanced applications, particularly in emerging optoelectronic technologies where their unique combination of optical, electronic, and chemical properties offers distinct advantages [3,4].

Melanins constitute a heterogeneous family of high-molecular-weight pigments derived from the oxidative polymerization of phenolic and indole compounds [5,6]. The classification of melanins into five major types—eumelanin, pheomelanin, neuromelanin, allomelanin, and pyomelanin—provides a framework for understanding the chemical and functional diversity of these remarkable biopolymers [7]. Each type exhibits unique structural features and properties that reflect its specific biosynthetic pathways and biological functions.

Among the various melanin types, eumelanin stands out for its stability, biocompatibility, and functional properties, making it particularly attractive for technological applications [8,9]. The complex structure of eumelanin remains a subject of debate. However, a general structural model consists of three levels of aggregation of oligomers of 5,6-dihydroxyindole (DHI), indole-2-carboxylic acid (ICA), and 5,6-dihydroxyindole-2-carboxylic acid (DHICA), which form an extended conjugated system responsible for its characteristic broadband UV-visible absorption and electronic properties [10,11,12].

Recent advances in natural product chemistry have revealed the remarkable potential of eumelanins in optoelectronic applications, where their unique properties enable novel functionalities not achievable with conventional synthetic materials [13,14]. The ability of eumelanins to exhibit mixed electronic-ionic conductivity, hydration state-dependent electrical properties, and broad-spectrum UV absorption has opened new avenues for applications in solar energy conversion, optical materials, and electronic devices [3,15]. These discoveries have transformed eumelanins from simple pigments to sophisticated functional materials with demonstrated utility in cutting-edge technologies [16,17].

The traditional sources of melanin, including cuttlefish ink and mammalian tissues, present limitations in terms of sustainability, scalability, and consistency [18,19]. Plant-derived melanins offer compelling advantages, including renewable feedstock, reduced environmental impact, and potentially unique chemical compositions that may enhance functionality [20,21]. L-DOPA is a crucial precursor in the eumelanin biosynthesis pathway [22]. *Mucuna* seeds have become a promising source due to their exceptionally high L-DOPA content.

Extensive studies have conclusively demonstrated that the synthesis of L-DOPA in *Mucuna pruriens* is carried out by polyphenol oxidase (PPO), rather than tyrosine hydroxylase or cytochrome P450 enzymes. This conclusion is supported by enzymatic inhibitor studies and in-gel activity assays, which have validated the central role of PPO in this metabolic pathway [23]. Recent genomic analysis of *Mucuna pruriens* has revealed the presence of 21 genes encoding polyphenol oxidase, showing the critical importance of this enzyme in L-DOPA biosynthesis [24]. Notably, PPO expression is particularly high in young stems and leaves of the plant [25].

*Mucuna ceniza*, a leguminous plant native to the tropical regions of México, may represent an outstanding source for sustainable eumelanin production when cultivated under controlled organic conditions [26,27]. A recent characterization of *M. ceniza* seed extracthas revealed L-DOPA content of up to 56% by weight, representing one of the highest concentrations achievable through sustainable extraction methods [28]. The standardized cultivation of *Mucuna* plants, under organic conditions, without the use of chemicals or pesticides, ensures consistent quality and environmental sustainability, while providing the high-quality precursor material essential for eumelanin biotransformation.

The bioactive properties of *Mucuna* species extend beyond their utility as L-DOPA sources, with recent studies demonstrating significant antioxidant, neuroprotective, and anti-inflammatory properties [29,30,31,32]. The L-DOPA in the extract can polymerize to form eumelanin, and the bioactive compounds present in the extract can probably copolymerize or become trapped within the eumelanin matrix which, in theory, could contribute to enhancing their final properties [33,34,35]. The demonstrated ability of *M. pruriens* L-DOPA extracts to prevent depression-like behaviors and reduce oxidative stress through mechanisms involving decreased lipid peroxidation and nitrite/nitrate levels, as well as increased glutathione concentrations, provides valuable insights into the antioxidant properties of this extract [36,37].

The development of standardized extraction protocols using green chemistry approaches has enabled the production of high-quality L-DOPA extracts suitable for biotechnological applications [38]. The use of acetic and citric acids as extraction solvents, combined with lyophilization for product stabilization, offers a sustainable alternative to harsh chemical extraction methods while preserving the integrity of the L-DOPA precursor and its associated bioactive compounds. This approach aligns with current trends toward environmentally responsible natural product processing, ensuring the preservation of functional properties essential for advanced applications [39].

The biotechnological production of melanin from standardized plant extracts involves the controlled oxidative polymerization of L-DOPA under optimized conditions [40]. This process can be systematically optimized through careful manipulation of pH, temperature, aeration, and agitation parameters in stirred-tank bioreactor systems [41].

Comprehensive analytical characterization plays a crucial role in natural product chemistry, particularly for complex biopolymers like melanin, where structure-property relationships determine functional performance [42]. State-of-the-art spectroscopic methods, including UV-visible, FTIR, Raman, and NMR spectroscopy, provide detailed insights into molecular structure, functional groups, and electronic properties [2,43]. These analytical approaches, combined with elemental analysis and chemical characterization, enable definitive identification and quality assessment of natural melanins while ensuring reproducibility and standardization.

The present study addresses the critical need for a comprehensive analytical characterization of eumelanin produced from standardized extracts of *M. ceniza* seeds achieved through the systematic application of analytical methods. Our objectives include the use of organically grown and standardized *M. ceniza* seeds and high-quality L-DOPA extracts as starting materials, the optimization of biotechnological eumelanin conditions using stirred tank bioreactor technology, comprehensive spectroscopic characterization using UV-Vis, FTIR, Raman, and NMR spectroscopy, and elemental analysis for the definitive identification and classification of produced eumelanin, as well as presenting evidence of its proven optoelectronic applications.

This work can make a significant contribution to the field of natural product chemistry by providing the first detailed analytical characterization of eumelanin from standardized *M. ceniza* seed extracts, establishing reproducible methods for quality assessment, and demonstrating the potential for sustainable biotechnological production of high-value functional materials.

## 2. Results

### 2.1. Standardized Melanin Preparation

The standardized L-DOPA extract obtained from organically cultivated *M. ceniza* seeds demonstrated quality and consistency over ten years, with an L-DOPA content of 56 ± 2% (*w*/*w*) maintained across multiple extraction batches [28]. For vegetal eumelanin obtention, the process optimization using this standardized extract achieved eumelanin production rates of 1526.23 ± 10.78 mg L^−1^ h^−1^ under optimal conditions (pH 11, 600 rpm agitation, 2.5 vvm aeration, 26 °C), representing high eumelanin production rates ever reported for plant-derived systems [44,45,46]. TLC followed the conversion from L-DOPA to eumelanin, with the absence of the L-DOPA band indicating their conversion was complete (Figure S1).

**Figure 1:**
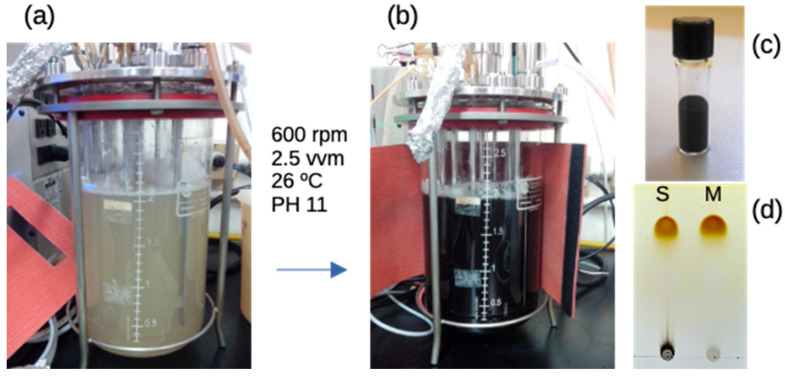
Process of melanin production in a bioreactor. The L-DOPA extract (**a**) is subjected to bioreactor conditions to produce melanin (**b**). The product is then washed and dried to form a powder (**c**). The melanin from *Mucuna* (M) is evaluated in TLC compared to the synthetic (S) standard melanin (M = *Mucuna* melanin) (**d**).

The optimization process involved a systematic evaluation of critical parameters affecting eumelanin formation from the complex plant extract matrix. As proof of concept, we initiated with the conversion of 0.1 g of commercial L-DOPA (Sigma Co., Burlington, MA, USA) into 1 L of solution in the reactor. At the end of the process, 0.0915 g of melanin was recovered, corresponding to a yield of 91.5%. The temperature of 26 °C was found to be optimal for maintaining eumelanin conversion [47]. The alkaline pH conditions (pH 11) facilitated the oxidative polymerization of L-DOPA while maintaining the stability of the bioreactor system and ensuring complete substrate conversion (Figure 1). The TLC analysis of *Mucuna* eumelanin revealed a single, brown-colored spot with an Rf value of 0.64, identical to that of synthetic melanin (Figure 1d). This finding is consistent with previous reports on natural melanin extracts [48]. Standard melanin tends to show higher polymerization, possibly accounting for the black/gray spot at the bottom of the TLC plate. The large brown/yellowish spots at the top may correspond to the expected migration of non-polar eumelanin fractions or oligomers. These are composed of DHI and 5,6-indolequinone building blocks, which form dimers to hexamers in π-stacked assemblies [12].

### 2.2. Elemental Analysis of Mucuna ceniza Melanin

The results of the elemental analysis of *Mucuna* eumelanin powder show a typical pattern similar to that of other vegetal eumelanins [2,49]. With the C/N relationship (~4.05) and the nitrogen content of (11.85%), as is typical of melanins with some protein content (Table 1).

The difference with theoretical values suggests a significant incorporation of amino and hydroxyl groups and a more complex structure related to the theoretical eumelanin model. The resultant empirical formulae can be approached to C_48_H_74_N_10_O_25_S_0.01_. The few contents of S in the sample may indicate that it is eumelanin, unlike pheomelanins, which generally have more sulfur content [50].

### 2.3. Spectroscopic Characterization and Structural Authentication

#### 2.3.1. UV-Visible Absorption Spectrum

**Figure 2:**
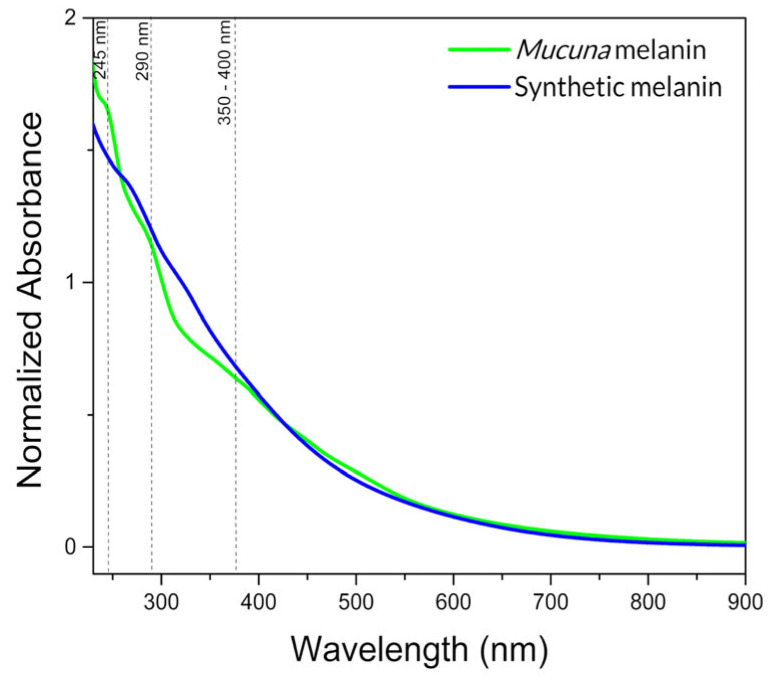
Normalized UV-visible spectra of melanins.

UV-visible spectroscopic analysis shows the typical monotonic decrease in absorbance for eumelanin-like materials [51], (Figure 2). A satisfactory linear correlation between the logarithm of the absorbance and wavelength for *Mucuna* and synthetic melanin (Figure S2) was obtained. This absorbance profile is a diagnostic feature that distinguishes eumelanin from other natural pigments [52]. The maximum peak absorption at 225–290 nm may correspond to the nitrogenated compound content of biological eumelanins, and the general pattern is characteristic of an amorphous and geometrically disordered polymeric biomaterial, such as eumelanin from diverse sources [33,53].

#### 2.3.2. FTIR Spectroscopy Analysis

**Figure 3:**
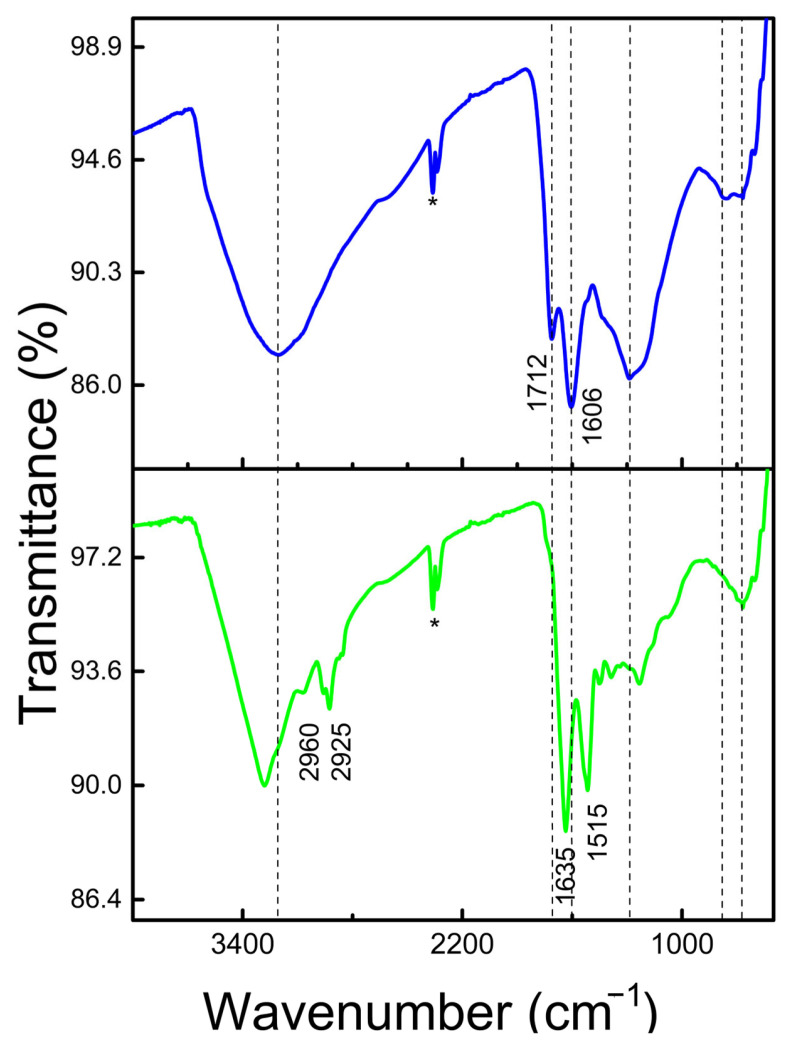
FTIR spectra of melanins. Synthetic melanin (blue) and *Mucuna* melanin (green). The two peaks marked with an asterisk indicate noise generated by the equipment.

The FTIR spectroscopy shows molecular vibrations when infrared radiation interacts with chemical bonds. In eumelanins, these vibrations correspond mainly to aromatic, carbonylic, and hydroxylic functional groups [50]. Eumelanins show a relatively complex pattern due to their heterogeneous composition. The broad region of ~3400–3200 cm^−1^ corresponds to -OH and N-H stretches of phenolic, carboxylic, and indole moieties (Figure 3), while the absorption bands at 2960 and 2925 cm^−1^ are attributed to aliphatic C–H stretching vibration [54]. The *Mucuna* eumelanin spectrum shows a clear and intense band (1635 cm^−1^) corresponding to carboxylate groups [55]. The origin of this signal may lie in the final step of eumelanin purification, where it is washed with a NaOH solution. The presence of the base can deprotonate the carboxylic acids present in eumelanin, resulting in the formation of carboxylates. On the other hand, in synthetic melanin, this band shifts to 1712 cm^−1^, suggesting the existence of carboxylic acids in the latter.

Other authors assign the band at 1635 cm^−1^ with the C=C stretching within the indole ring, while the features at 1515 and 1454 cm^−1^ are associated with C–H deformation and coupled vibrational modes [56]. The broad region spanning 1000–1300 cm^−1^ can be ascribed to C–N stretching vibrations of the indole moiety [56]. Signals below 1000 cm^−1^ indicate the presence of aromatic ring structures, potentially with substitution patterns. Additionally, the aliphatic C–C stretching observed at 1453 cm^−1^ may reflect the presence of protein residues closely associated with the eumelanin matrix [57]. It is observed that the absence of a peak between 700 and 600 cm^−1^ (Figure S3), typical of sulfur groups in pheomelanins [58], indicates again that this compound is probably eumelanin. Finally, the differences between the two spectra are attributed to the different chemical environments around the functional groups. The two peaks marked with an asterisk correspond to noise equipment; however, these signals do not affect the discussion mentioned above, as there are no significant bands in this region.

#### 2.3.3. Raman Spectroscopy Analysis

**Figure 4:**
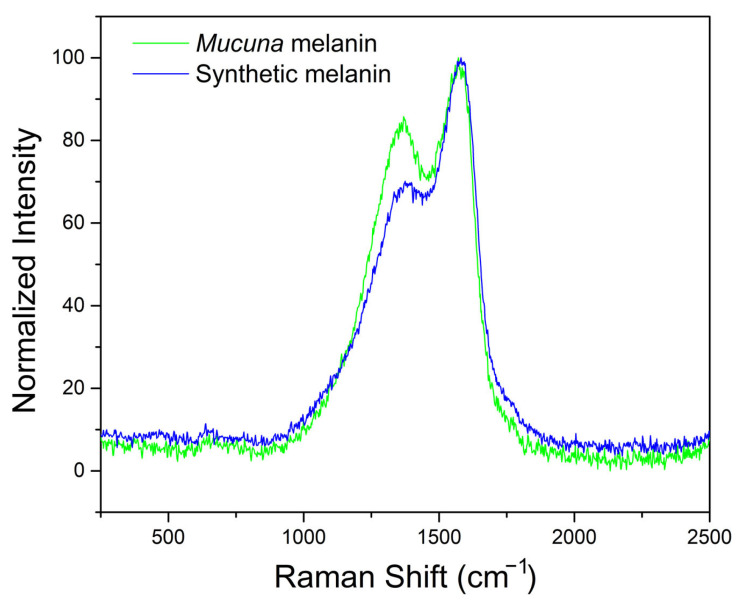
Raman spectral characterization of melanins.

Raman spectroscopy is based on the phenomenon of inelastic dispersion of monochromatic light (laser) when it interacts with vibrational molecular modes, particularly in conjugated aromatic rings, such as those that form the polymeric structure of eumelanins. This technique provided complementary structural information to FTIR. It confirmed the eumelanin identity through characteristic vibrational bands at 1380 and 1580 cm^−1^ (Figure 4). The prominent peak in synthetic melanin at 1580 cm^−1^ corresponds to the aromatic rings’ breathing modes and C=C stretching vibrations, characteristic of the indole-based polymer backbone, typical of eumelanin structures [59]. The 1380 cm^−1^ peak in eumelanin Raman spectra originates from linear stretching of C-C bonds within aromatic rings, with contributions from C-H vibrations in methyl and methylene groups [60].

In the *Mucuna* eumelanin, a more prominent peak at 1380 cm^−1^ is observed, which could indicate a more complex conjugated structure attributable to a different subunit composition compared with the synthetic one. The characteristic profile of the band within the 550–1200 cm^−1^ region indicates a high abundance of DHI units. In contrast, the absence of significant spectral broadening in the 1650–2300 cm^−1^ region suggests a high DHI:DHICA ratio in the *Mucuna* eumelanin [60]. This analysis confirms the identity as eumelanin when compared to other studies with natural melanins [61,62].

#### 2.3.4. NMR Spectroscopy Analysis

**Figure 5:**
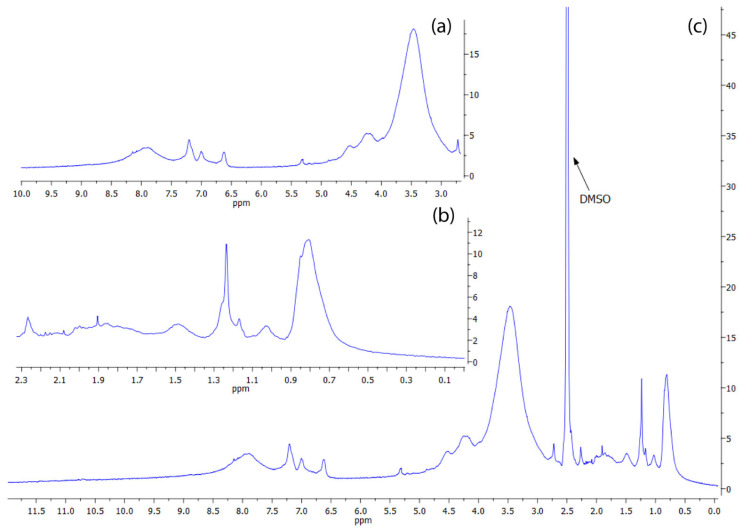
^1^H-NMR Spectral Characterization of *Mucuna*’s eumelanin. For a better appreciation of the absorption peaks of the overall spectrum (**c**), this was divided into the insets (**a**,**b**) for before and after the DMSO signal peak (2.5 ppm).

The ^1^H-NMR spectral chemical shift (δ) is represented as parts per million (ppm) to be comparable across different instruments. In Figure 5, the ^1^H-NMR spectrum of *Mucuna* eumelanin is presented. Due to the chemical complexity of the eumelanin structure and its supramolecular interactions, a spectrum with broad signals is obtained. The solvent residual peak (DMSO) is present at 2.5 ppm. For a better appreciation, the spectrum is divided into insets 5a and 5b, alongside the DMSO peak.

The broad resonance observed around 8.0 ppm was assigned to hydroxyl (–OH) groups attached to aromatic rings (Figure 5a). Additionally, the signals appearing between 6.5 and 7.3 ppm were attributed to aromatic protons of indole and/or pyrrole moieties. The signals in the region of 4.5–5.4 ppm were consistent with vinylic protons (C=C–H) adjacent to nitrogen and/or oxygen atoms [63]. The spectrum shows a water signal at 3.3 ppm, common for DMSO-d_6_ water absorption that overlaps with the resonances detected between 3.2 and 4.2 ppm, ascribed to methylene or methyl groups bonded to heteroatoms such as nitrogen or oxygen (CH_2_OH) [49], which is consistent with hydroxylated side chains or partially oxidized aliphatic components within the eumelanin polymer matrix.

The signals within the range of 1.3–2.3 ppm were attributed to the presence of an NH group linked to indole [49]. Finally, alkyl groups in eumelanins (0.5–2.5 ppm) correspond to the visible peaks in the aliphatic region (Figure 5b). The multiple peaks in this zone are likely contributed to by methylene bridges and the incorporation of lipids and fatty acids during eumelanin production, as well as residual nitrogenated compounds [2]. No peaks corresponding to carboxylic acid protons (typically expected at 10–12 ppm) were detected, possibly due to hydrogen-deuterium exchange, which is attributable to DHICA units. The comparison of the ^1^H-NMR spectrum of *Mucuna* eumelanin with that of *Catharsius molossus* L. melanin (an entirely biological sample) reveals remarkable similarities (Figure S4), indicating that the two types of melanins might have the same structure [57].

Additionally, the liquid ^13^C-NMR spectrum displayed only a limited number of resonances (Figure S5). The most intense signal appeared at 171 ppm, consistent with carbonyl carbons from peptide bonds, carboxyl or amide side-chain groups, and carbonyl functionalities associated with eumelanin quinones [63].

## 3. Discussion

Melanin represents one of the most ubiquitous and functionally diverse biopolymers found across living organisms, from microorganisms to higher animals and plants. Their intense research is partly due to the search for new sources of materials with desired characteristics [64]. Social trends demand sustainable production methods [65]. Therefore, the availability of biomaterials is relevant for future societies. Particularly, eumelanins have long been a promising biomaterial due to their optical and electronic properties [66]. There are numerous reviews and proof-of-concepts in their applications [67,68,69]; however, to date, there is no industry based on eumelanins due to their scarcity and prohibitively high cost [18,70].

The process of producing eumelanin from *Mucuna pruriens* seeds takes place in two main phases. The initial stage involves an extraction process that yields an extract enriched in L-DOPA, with a 20% yield, equivalent to 200 kg of extract per ton of processed seeds. Subsequently, the second stage of biotransformation yields 50 kg of eumelanin per ton of seed, representing a final yield of 5% relative to the initial seed mass. The total cost of eumelanin production, including both phases, has been estimated at USD $500 per kilogram. This calculation includes a 10×multiplier to reflect the complexity of the biotechnological process. It incorporates the cost of raw materials (USD $2500 per ton of seeds, based on maximum prices on the Indian international market), as well as the costs inherent in extraction, biotransformation, final purification, labor, depreciation, and direct and indirect operating expenses.

In terms of market value, commercial-grade synthetic eumelanin shows significant price variability depending on the supplier and purity level, underscoring the high value of this biomaterial. For example, synthetic melanin (Reference M8631) offered by Sigma-Aldrich for research use costs USD $648 per gram, equivalent to USD $648,000 per kilogram. Other suppliers, such as Spectrum Chemical and Biosynth, offer similar products with prices ranging from USD $707,700 to USD $1,655,250 per kilogram. This market valuation contrasts with the estimated production cost, which emphasizes the upside of the biotechnological route developed.

Here, it is demonstrated that eumelanin can be obtained in a renewable manner at a cost that could be competitive for industrial applications. The *Mucuna* plants from which it is extracted are leguminous plants that provide multiple environmental benefits through their cultivation: contributing to soil recovery with their biomass [71], enhancing soil nutrition by fixing and incorporating nitrogen in their roots through symbiotic bacteria [72], and protecting small species with their abundant foliage. The plant is also used as a cover crop and intercrop, mainly with maize [73].

Recent research has revealed that eumelanin exhibits a hierarchical organization, with oligomeric sheets forming proto-particles that subsequently aggregate into larger, anti-spherical particles. This structural organization contributes to eumelanin’s unique optical and electronic properties, making it an attractive material for bioelectronics applications [74]. The polymer’s ability to conduct both electrons and ions, combined with its biocompatibility, has led to its investigation in organic electronics and biomedical devices [75]. The elemental analysis and the more common spectroscopic analyses presented here confirm that the eumelanin extracted in this way from *Mucuna pruriens* var. ceniza very probably corresponds to eumelanin.

Numerous reports have documented the beneficial biological activity of *Mucuna pruriens*, primarily in the seeds [76,77,78]. In particular, the L-DOPA-enriched extract from which eumelanin is obtained has been shown to prevent depression-like behaviors after a mild traumatic brain injury, with evidence of reducing oxidative stress, diminishing lipid peroxidation, and decreasing nitrite and nitrate levels, while increasing reduced glutathione in specific brain areas [36].

On the other hand, the eumelanin described here has already shown potential applications in optoelectronic technologies, validating the technological relevance of its comprehensive characterization and sustainable production approach. In one of the first studies, eumelanin/PSi composites demonstrate their potential for increasing radiative recombination centers at eumelanin-PSi heterojunctions, as indicated by an increase in silicon-hydrogen (Si–H) and silicon-oxygen (Si–O) defects, as confirmed by Fourier Transform Infrared (FT-IR) spectroscopy. A remarkable decrease in microsecond-time components in pristine PSi toward nanosecond decay times, accompanied by eumelanin, indicates an increase in radiative recombination centers. Scanning electron microscopy (SEM) reveals the distribution of small grains of eumelanin on the surface of the PSi, progressing to the formation of a thin eumelanin film [79]. In a second study, eumelanin increases the energy efficiency of a solar panel by 3% (resulting in a total increment in solar cell conversion efficiency of approximately 44%), which depends on the uniform distribution of the eumelanin/PSP colloidal solution over the glass [80].

A third study demonstrated that eco-friendly and low-cost materials (eumelanin + FeOx) can be utilized to fabricate supercapacitors capable of operating optimally at high temperatures (70 °C), owing to the enhancement of ion conductivity through the electrodes. This device maintained a high capacitance retention of 84–88% after 5000 cycles of charge–discharge or 1000 bending cycles. Raman and XPS spectroscopies revealed a higher presence of oxygen-vacancy defects and Fe^3+^/Fe^2+^ species on electrodes made with eumelanin and FeOx [81].

The sustainable production approach developed in this study addresses critical environmental and economic considerations for the commercial production of eumelanin. The organic cultivation methods ensure environmental sustainability while maintaining consistent product quality over the course of 10 years of annual production. The method proposed here combines the use of a renewable and organic raw material (*M. ceniza* seeds) with an initial extraction process using organic acids, followed by biotransformation in a bioreactor under ambient conditions. The pH is controlled using a 0.1 N NaOH solution, ensuring minimal environmental impact. The biotransformation of the seed extract into eumelanin relies on auto-oxidation, which can occur without the need for external catalysts, as has already been demonstrated with L-DOPA; still, it took a week [82]. Further research and characterization of this eumelanin are pending for various applications.

## 4. Materials and Methods

### 4.1. Plant Material and Standardized Cultivation

*Mucuna ceniza* seeds were obtained from plants cultivated under organic conditions in Tepecoacuilco, State of Guerrero, México (18°18′0″ N, 99°29′0″ W). The cultivation was conducted on farmland fertilized exclusively with cattle manure, without the use of synthetic chemicals or pesticides, thereby ensuring compliance with organic certification standards and promoting environmental sustainability. The cultivation process was entirely dependent on the natural rain-based season. In our practice, we cultivate approximately 2500 plants/ha, with a productivity of around 1 ton/ha. The plant takes 20 weeks (July to November). Each plant produced an average of 125 pods, with four seeds per pod and an individual seed weight of 0.8 g, resulting in a total yield of approximately 400 g per plant. These parameters are consistent with productivity data reported in Costa Rica [83]. Seeds were harvested at optimal maturity, cleaned to remove debris, and stored under controlled conditions (4 °C, 60% relative humidity) to preserve L-DOPA content and prevent degradation of bioactive compounds.

### 4.2. Sustainable L-DOPA Extraction and Standardization

The standardized extraction procedure involved grinding clean, dried *M. ceniza* seeds through a 2 mm mesh, thereby increasing the surface area for extraction while maintaining cellular integrity and preserving bioactive compounds. A total of 100 g of milled, dried seeds were suspended in 1 L of an extraction solution composed of 0.3% acetic acid and 0.1% citric acid (1:10 *w*/*v*) at room temperature for 24 h under continuous agitation (100 rpm). The extract was filtered, concentrated under reduced pressure, and lyophilized to obtain a stable powder enriched in L-DOPA [28]. The yield of freeze-dried powder from the L-DOPA-enriched extract was 20% (i.e., 20 g of powder was obtained from 100 g of ground seeds).

### 4.3. Eumelanin Conversion Optimization in Bioreactor

Eumelanin production was conducted in a 3 L stirred-tank bioreactor (Applikon, Vlaardingen, The Netherlands) using 38 g/L of the standardized L-DOPA extract as starting material in a buffer composed of 0.1 M sodium carbonate (Na_2_CO_3_) and sodium bicarbonate (NaHCO_3_) in a 5:1 molar ratio, which was used to maintain the pH at 11. The bioreactor system was equipped with two six-blade Rushton turbines for efficient mixing and mass transfer, which are essential for the oxidative polymerization reactions that lead to eumelanin formation.

Temperature control was achieved using a jacketed vessel connected to a thermostatic bath, which maintained a temperature of 26 °C, based on preliminary optimization studies. The pH was maintained at 11.0 using the automated addition of 0.1 M NaOH solution. Aeration was provided at 2.5 vvm (volume of air per volume of medium per minute) through a sparger system to ensure adequate oxygen supply for the oxidative polymerization process.

The biotransformation process was monitored through regular sampling and analysis of L-DOPA consumption by TLC (visualized with iodine staining) and pH stability (Figure S1).

### 4.4. Eumelanin Isolation and Purification

Following biotransformation, the eumelanin product was isolated through a multi-step purification process designed to remove residual substrate, salts, and other impurities while preserving the polymer structure. The bioreactor contents were acidified to a pH of 3.0 (using approximately 100 mL of 1 N HCl) to precipitate the eumelanin, which was then collected by centrifugation at 10,000× *g* for 15 min. The crude eumelanin was washed repeatedly (5× with distilled water until the washings were colorless, followed by acid washing (0.5 L, 0.1 N HCl) to remove metal ions and alkaline washing (0.5 L, 0.1 M NaOH) to remove low-molecular-weight impurities. The purified eumelanin was lyophilized (Labconco, Kansas City, MO, USA) and stored under desiccated conditions for analytical characterization. The yield was around 25% of eumelanin powder relative to the L-DOPA extract. The overall process yielded 5% eumelanin based on the initial weight of seed, meaning 5 g of eumelanin was obtained from 100 g of seeds. 

### 4.5. Elemental Analysis

The elemental composition (C, H, N, S) was determined using a CHNS elemental analyzer (PerkinElmer 2400 Series II, Waltham, MA, USA) with acetanilide as the standard. Samples were combusted at 925 °C in a pure oxygen atmosphere, and the combustion products were analyzed by gas chromatography with thermal conductivity detection.

### 4.6. Thin Layer Chromatography

Eumelanin samples (20 mg) were dissolved in 1 mL DMSO, sonicated for 5 min (Branson, Emerson Electric Co., St. Louis, MO, USA), and applied to silica gel TLC plates (Merck 60 F254, Darmstadt, Germany). The mobile phase used for development consisted of n-butanol, acetic acid, and water in a ratio of 70:20:10 (*v*/*v*/*v*), and the eumelanin spots were visualized using iodine staining. Synthetic L-DOPA and melanin were purchased from Sigma Co. (Burlington, MA, USA).

### 4.7. Analytical Characterization Methods

#### 4.7.1. UV-Visible Spectroscopy

UV-visible spectra were recorded using a double-beam spectrophotometer (Shimadzu UV-2600, Nakagyo-ku, Kyoto, Japan) in the wavelength range of 200–900 nm. 20 mg of synthetic (from Sigma, prepared by oxidation of tyrosine with hydrogen peroxide) and vegetal eumelanin samples were dissolved in 1 mL DMSO. The mixture was vigorously shaken for 2 min and then ultrasonicated for 5 min. For the analysis of the sample, it was diluted 1:10 with distilled water. The baseline correction was performed using the solvent blank, and the spectra were normalized to the minimal absorbance for comparative analysis.

#### 4.7.2. FTIR Spectroscopy

Eumelanin samples were analyzed using mid-infrared spectroscopy with attenuated total reflectance (ATR) equipped with a diamond crystal (Thermo Scientific Nicolet iS50, Waltham, MA, USA). In particular, 5 mg of eumelanin was placed directly on the ATR crystal with optimal pressure applied to ensure spectral uniformity. Spectra were obtained by averaging 64 scans at a 4 cm^−1^ resolution over the range of 4000–500 cm^−1^, under ambient conditions. The signals observed around 2300 cm^−1^ (highlighted with an asterisk in Figure 3) are attributed to equipment noise. This noise is a constant feature present in all recorded spectra and does not correspond to any chemical vibrations typical of eumelanin materials. This signal appears in a region devoid of significant molecular vibrations and therefore does not interfere with the interpretation of other spectral features.

#### 4.7.3. Raman Spectroscopy

Raman spectra were acquired using a DXR3xi Raman imaging microscope (Thermo Scientific, Waltham, MA, USA) equipped with a 10× objective and a confocal pinhole set to 25 μm in diameter. The excitation wavelength was 532 nm with power settings of 2–5 mW to prevent sample degradation. Spectra were acquired over the range of 100–3500 cm^−1^ with a resolution of 4 cm^−1^.

#### 4.7.4. NMR Spectroscopy

^1^H and ^13^C-NMR spectra were recorded on a Bruker Avance spectrometer operating at 300 MHz, using DMSO-d_6_ as solvent and tetramethylsilane (TMS) as the internal standard. Ten milligrams (10 mg) of the melanin sample were dissolved in 0.8 mL of deuterated dimethyl sulfoxide (DMSO-d_6_). The resulting solution was sonicated before analysis.

### 4.8. Statistical Analysis

All experiments were performed in triplicate, and results are expressed as mean ± standard deviation. When applied, statistical significance was evaluated using one-way ANOVA with Tukey’s post hoc test, with *p* < 0.05 considered statistically significant. Data analysis was performed using GraphPad Prism 9.0 software.

## 5. Conclusions

The decade-long organic cultivation of *Mucuna pruriens* var. ceniza and standardized extraction protocols have established a reliable supply chain for high-quality starting material, yielding L-DOPA concentrations of 56% (*w*/*w*)—among the highest reported for sustainable extraction methods. This approach not only preserves the bioactive compounds responsible for the demonstrated antioxidant and neuroprotective properties of *Mucuna* extracts but also enables the optimized biotechnological production process that sets a new benchmark for plant-derived eumelanin synthesis, achieving production rates of 1526.23 ± 10.78 mg L^−1^ h^−1^ in the bioreactor.

Comprehensive analytical characterization, including elemental composition analysis, provided evidence of the more probable classification of the product as eumelanin. Multi-technique spectroscopic validation, using UV-visible, FTIR, Raman, and NMR spectroscopy, confirmed the production of eumelanin with structural characteristics equivalent to those of commercial synthetic standards.

The integration of sustainable production methodology with reported optoelectronic capabilities positions this eumelanin as a high-value natural product that addresses critical environmental and economic considerations essential for commercial viability. The potential scalability and reproducibility of the process, combined with competitive production costs, establish a compelling case for potential industrial implementation. Ensuring the purity of eumelanin and identifying any metabolites embedded in its structure will be critical to guarantee a material suitable for specific biomedical applications.

This study not only validates *Mucuna ceniza* as a sustainable source for eumelanin production but also contributes to the development of bio-based, high-performance materials that can reduce our dependence on synthetic counterparts while potentially offering enhanced functionality. 

Future research should focus on exploring the full potential of this novel eumelanin in specific optoelectronic applications, including organic photovoltaics, biocompatible sensors, and energy storage devices. Additionally, further investigation into the structure-property relationships governing its performance characteristics will be important for maximizing its application-specific properties and unlocking its full commercial potential.

## Figures and Tables

**Table 1:** Elemental analysis. Results are the average of two production lots.

Element	Experimental ^a^	Predicted ^b^	Difference
C (%)	48.04 ± 0.15	51.25	−3.21
H (%)	6.14 ± 0.09	4.50	+1.64
N (%)	11.85 ± 0.01	7.93	+3.92
S (%) ^c^	0.03 ± 0.01	--	--
O (%) ^d^	33.94	35.82	−1.88

^a^ media ± SD (*n* = 2); ^b^ is calculated from the structure of typical eumelanin; ^c^ semi-quantitative determination; ^d^ calculated by difference.

## Data Availability

The original contributions presented in this study are included in the article/Supplementary Material. Further inquiries can be directed to the corresponding author(s).

## Supplementary Materials

The following supporting information can be downloaded at: https://www.mdpi.com/article/10.3390/ijms262110298/s1.

## Abbreviations

The following abbreviations are used in this manuscript:

DHI	5,6-dihydroxyindole
DHICA	5,6-dihydroxyindole-2-carboxylic acid
vvm	volume of air per volume of medium per minute
DMSO	Dimethyl sulfoxide
PSi	Porous silicon
PSP	Porous Silicon Powder
