# Supplementary material for: Sustainable Production and Characterization of Eumelanin from Organically Cultivated Mucuna ceniza Seeds: A High-Performance Biomaterial for Optoelectronic Applications
Source: Int J Mol Sci. 2025 Oct 23;26(21):10298. doi: 10.3390/ijms262110298 (PMC12608612; doi:10.3390/ijms262110298)
Supplement: Supplementary file 1 [file ijms-26-10298-s001.zip › ijms-3924348-supplementary.pdf]

Supplementary Materials

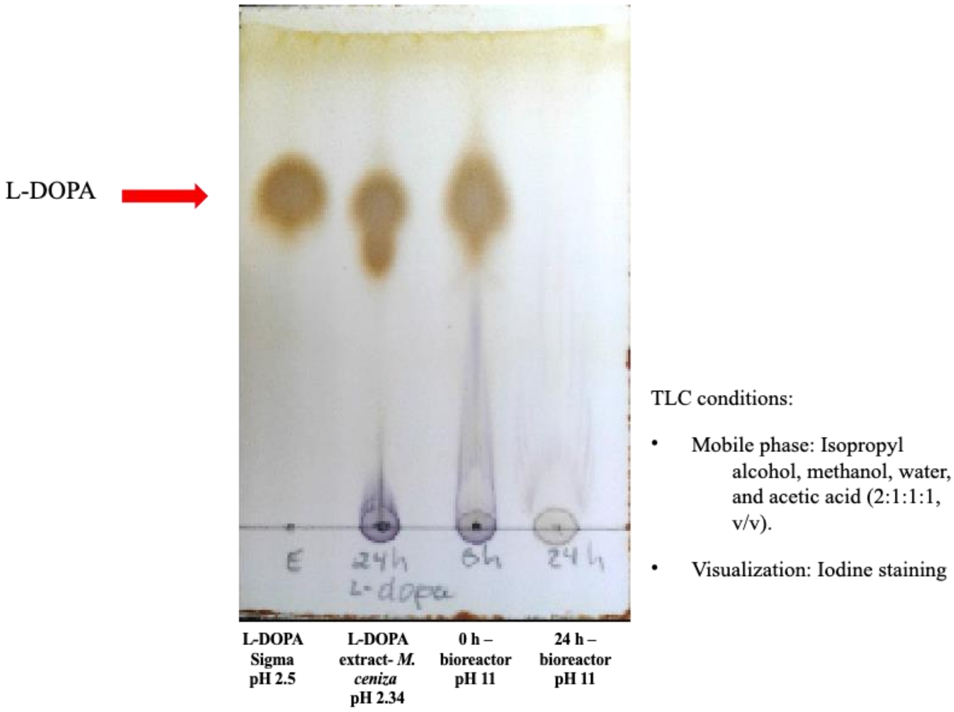

Figure S1. TLC followed the process of L-DOPA to eumelanin conversion.

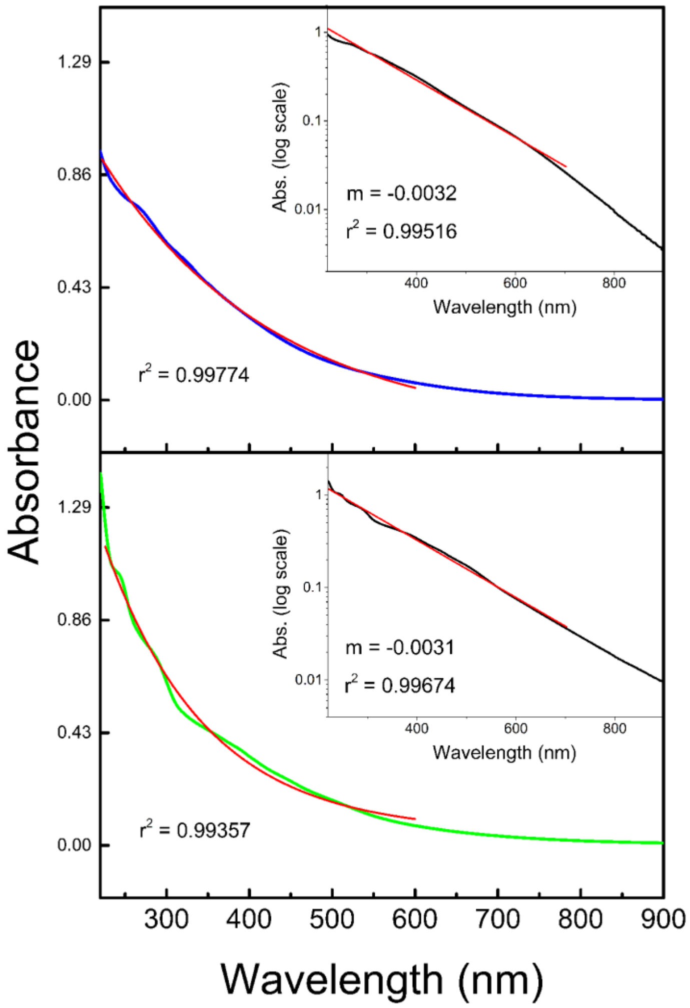

Figure S2. Absorbances as a function of wavelength. The inset shows the logarithm of the absorbance as a function of wavelength. The red lines represent the exponential and linear regression lines for *Mucuna* (green) and synthetic melanin (blue).

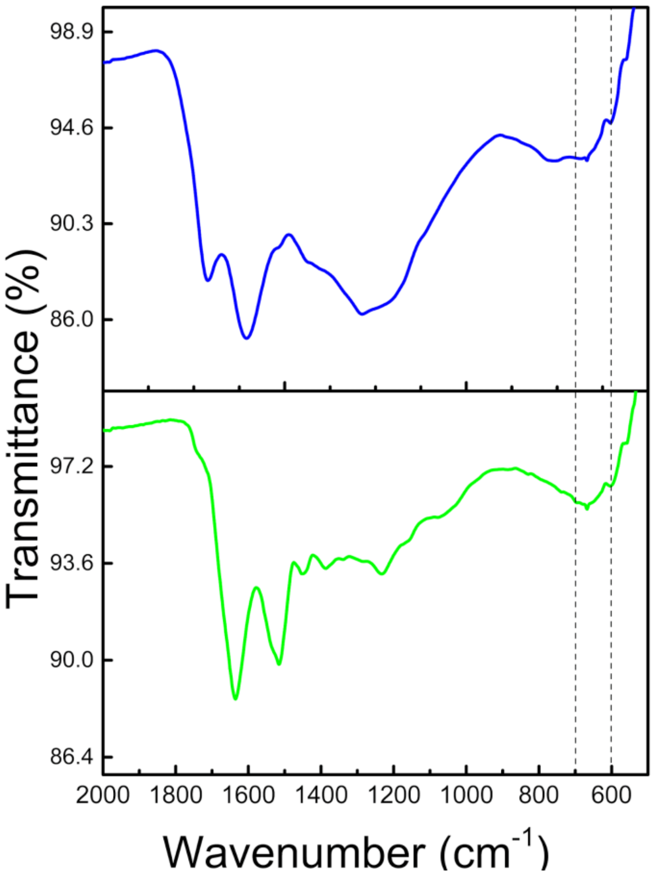

Figure S3. FTIR spectrum of *Mucuna* (green) and synthetic (blue) melanin. There is no presence of the C–S stretching vibration peak at 700–600 cm^{-1}, typical of pheomelanin.

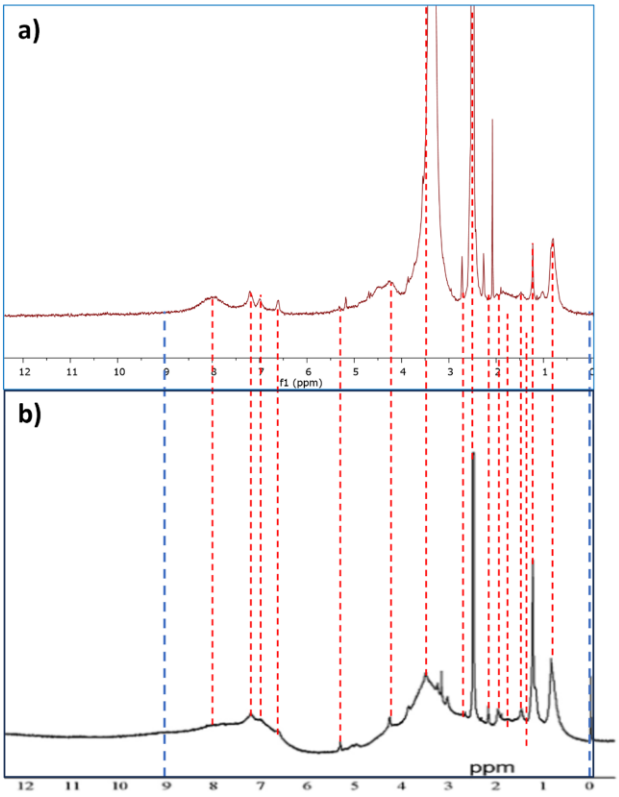

Figure S4. H-NMR spectra comparison of a) *Mucuna* melanin and b) *Catharsius molossus* L. melanin. Figure b) was adapted from [1].

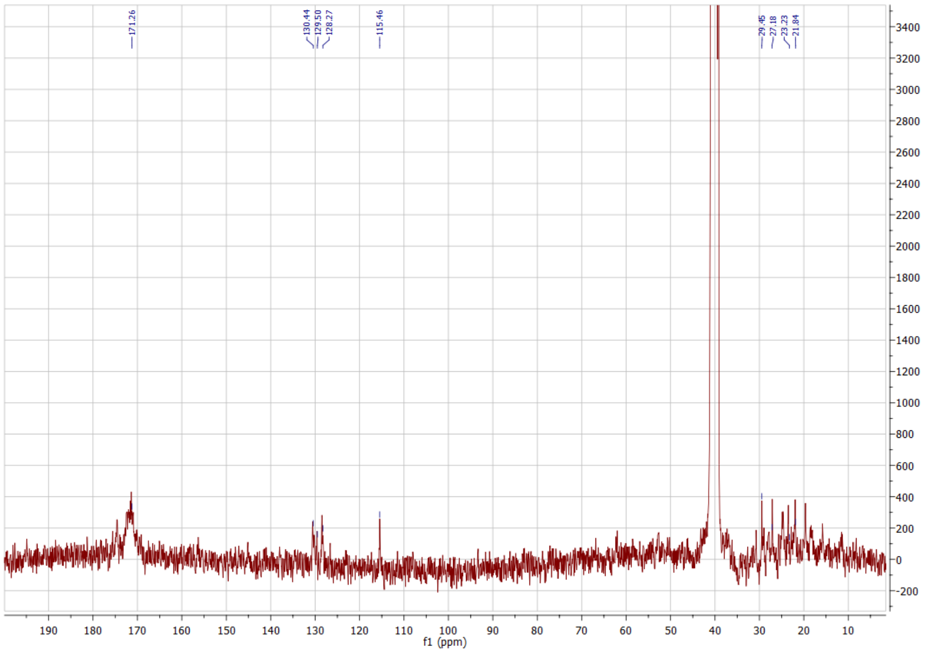

Figure S5. Liquid ^{13}C-NMR spectrum of *Mucuna* melanin. The signal at 40 ppm corresponds to DMSO. The peaks between 110–130 ppm could be generated by aromatic carbons, probably involved in indole or pyrrole systems. In contrast, the peaks of carbon atoms from methyl and methylene groups were detected within 10–40 ppm, as compared to the reference [2].

References

1. Xin, C.; Ma, J.; Tan, C.; Yang, Z.; Ye, F.; Long, C.; Ye, S.; Hou, D. Preparation of Melanin from Catharsius Molossus L. and Preliminary Study on Its Chemical Structure. *J. Biosci. Bioeng.* **2015**, *119*, 446–454, doi:10.1016/j.jbiosc.2014.09.009
2. Pralea, I.-E.; Moldovan, R.-C.; Petrache, A.-M.; Ilieș, M.; Hegheș, S.-C.; Ielciu, I.; Nicoară, R.; Moldovan, M.; Ene, M.; Radu, M.; et al. From Extraction to Advanced Analytical Methods: The Challenges of Melanin Analysis. *Int. J. Mol. Sci.* **2019**, *20*, 3943, doi:10.3390/ijms20163943.
